# Sex differences in the prevalence of radiographic findings of structural hip deformities in patients with symptomatic femoroacetabular impingement

**DOI:** 10.1093/jhps/hnab050

**Published:** 2021-06-22

**Authors:** Jun Zhou, Heath P Melugin, Rena F Hale, Bryant M Song, Kelechi R Okoroha, Bruce A Levy, Aaron J Krych

**Affiliations:** Department of Orthopedic Surgery, The First Affiliated Hospital of Soochow University, 296 Shizi St, Cang Lang Qu, Suzhou, Jiangsu, China; Department of Orthopedic Surgery, Mayo Clinic, 200 1st St SW, Rochester, MN 55905, USA; Department of Orthopedic Surgery, Mayo Clinic, 200 1st St SW, Rochester, MN 55905, USA; Department of Orthopedic Surgery, Mayo Clinic, 200 1st St SW, Rochester, MN 55905, USA; Department of Orthopedic Surgery, Mayo Clinic, 200 1st St SW, Rochester, MN 55905, USA; Department of Orthopedic Surgery, Mayo Clinic, 200 1st St SW, Rochester, MN 55905, USA; Department of Orthopedic Surgery, Mayo Clinic, 200 1st St SW, Rochester, MN 55905, USA

## Abstract

The purpose of this study was to determine the sex differences in the overall prevalence of radiographic femoroacetabular impingement (FAI) deformity patients presenting with hip pain and to identify the most common radiographic findings in male and female patients. A geographic database was used to identify patients between the age of 14 and 50 years with hip pain from 2000 to 2016. A chart and radiographic review was performed to identify patients with cam, pincer and mixed-type FAI. A total of 374 (449 hips) out of 612 (695 hips) male patients and 771 (922 hips) out of 1281 (1447 hips) female patients had radiographic features consistent with FAI. Ninety-four male hips (20.9%) and 45 female hips (4.9%) had cam type, 20 male hips (4.5%) and 225 female hips (24.4%) had pincer type and 335 male hips (74.6%) and 652 female hips (70.7%) had mixed type. The overall prevalence of radiographic findings consistent with FAI in male and female patients with hip pain was 61.1% and 60.2%, respectively. Mixed type was the most prevalent. The most common radiographic finding for cam-type FAI was an alpha angle >55°, and the most common radiographic finding for pincer-type FAI was a crossover sign. Male patients were found to have a higher prevalence of cam-type deformities, whereas female patients were found to have a higher prevalence of pincer-type deformities.

## INTRODUCTION

Femoroacetabular impingement (FAI) is a common cause of hip pain and is a known risk factor for hip osteoarthritis and total hip arthroplasty at a young age [1–9]. The diagnosis of FAI is based on clinical symptoms, physical examination findings and imaging abnormalities. Although there have been substantial advancements in FAI diagnostic imaging modalities such as computed tomography [10, 11] and magnetic resonance imaging [12, 13], the conventional radiograph remains the most common method in the initial evaluation of a patient with FAI [14, 15].

There are three types of FAI: cam, pincer and mixed. Cam-type FAI is characterized by an abnormal/aspheric morphology of the femoral head and pincer type is characterized by focal or global acetabular over-coverage. Mixed type consists of a combination of cam and pincer characteristics [4, 16–20]. Surgical and non-surgical treatment options are based on different types and severity of FAI; therefore, it is important to accurately identify the type of FAI [14]. Radiographic parameters include the crossover sign (COS), posterior wall sign (PWS), ischial spine sign (ISS), coxa profunda, protrusion acetabuli, lateral center edge angle (LCEA), Tönnis angle and alpha angle [7, 21–27]. Previous studies have described the radiographic findings of FAI in athletes [28–32], asymptomatic volunteers [33], adolescents [34] and a general population of patients with hip pain [35]. However, the prevalence of radiographic findings of FAI in patients with hip pain based on sex is understudied.

Therefore, the purpose of this study was 3-fold: (I) to determine the overall prevalence of radiographic FAI deformities in male and female patients presenting with hip pain, (II) to identify the most common radiographic findings in male and female patients with cam-type FAI and (III) to identify the most common radiographic findings in male and female patients with pincer-type FAI. This study involves a large cohort of patients with hip pain, which is unique. We are unaware of any prior study that reports the difference in prevalence based on the sex of FAI radiographic abnormalities in patients presenting to their physician with hip pain. Prior studies have evaluated asymptomatic patients in smaller cohorts. We hypothesized that the overall prevalence of radiographic findings consistent with FAI will be similar between males and females.

## METHODS

A geographic-based cohort study was performed with the Rochester Epidemiology Project (REP) database in Olmsted County, MN, USA, which had a population of 144 260 in 2010. The REP is medical record linkage system that provides access to the complete medical records for all residents of Olmsted County, regardless of the medical facility in which the care was delivered [36]. Institutional review board approval (IRB#: 17-l004959, IRB Modification #: Mod17-004959-02) was obtained, and the REP was used to identify all patients in a geographic area who presented to a physician with hip pain and had an initial diagnosis of an International Classification of Diseases, Ninth Revision or Tenth Revision, diagnostic code of hip pain, hip impingement or hip joint disorders between January 2000 and December 2016. Only patients aged between 14 and 50 years were included. The upper age limit is consistent with prior studies on FAI [7, 37]. Patients with a history of avascular necrosis, trochanteric bursitis, hip fracture, pelvic fracture, previous hip surgery and/or hip dislocation were excluded.

**Fig. 1.**
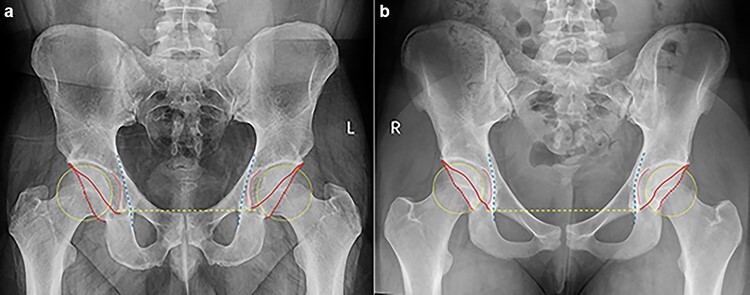
Standard anteroposterior pelvic views of normal hips in male (a) and female (b), a yellow dash line connecting bilateral inferior margins of pelvic teardrops represents a horizontal baseline, a yellow dot best-fit circle represents the right femoral head contour, a red dot represents the center of femoral head, a red line represents anterior acetabular rim, a red dash line represents posterior acetabular rim, a red dot curve represents acetabuli fossa and a blue dash line represents ilioischial line.

**Fig. 2.**
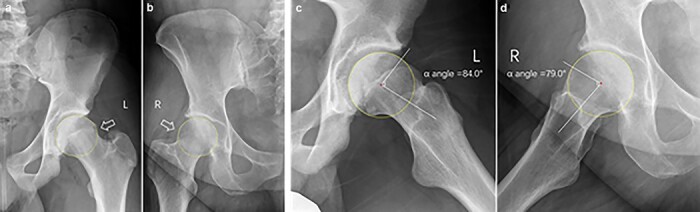
Typical pistol grip deformity in male (a, arrow) and female (b, arrow), and a yellow dot best-fit circle represents the femoral head contour; Alpha angle > 55° in male (c) and female (d), frog leg view.

**Fig. 3.**
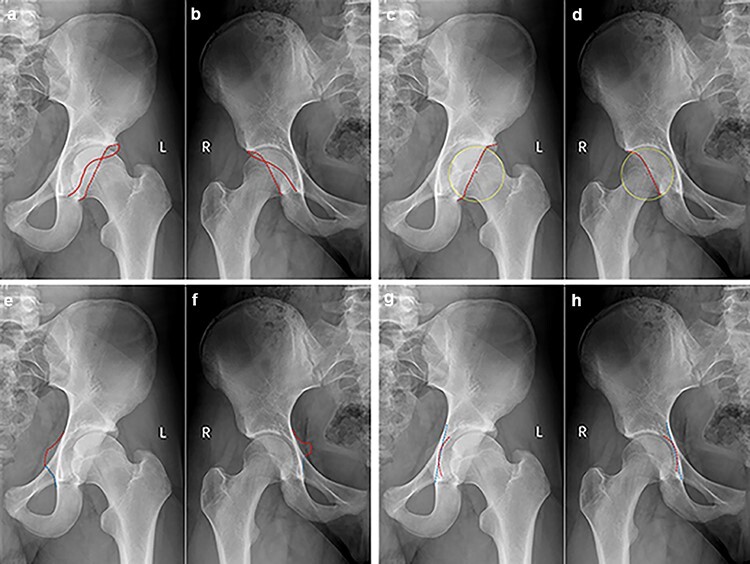
Cross-over sign in male (a) and female (b), red line represents anterior acetabular rim and red dash line represents posterior acetabularrim; Posterior wall sign in male (c) and female (d), yellow dot best-fit circle represents the femoral head contour, red point represents the center of femoral head and red dash line represents posterior acetabular rim; Ischial spine sign in male (e) and female (f), red dash line represents protrude ischial spine; and Coxa profunda in male (g) and female (h), red dash curve represents acetabuli fossa and blue dash line represents ilioischial line.

**Fig. 4.**
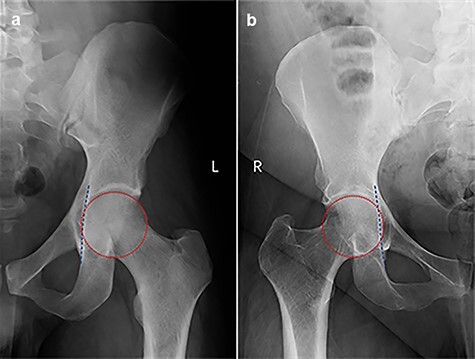
Protrusio acetabuli in male (a) and female (b), the best-fit circle represents the femoral head contour and the dashed line defines the ilioischial line.

**Fig. 5.**
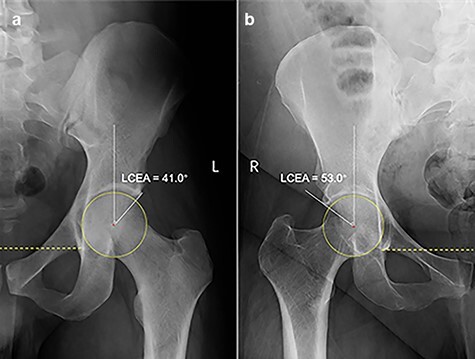
LCEA≥40° in male (a) and female (b).

**Fig. 6.**
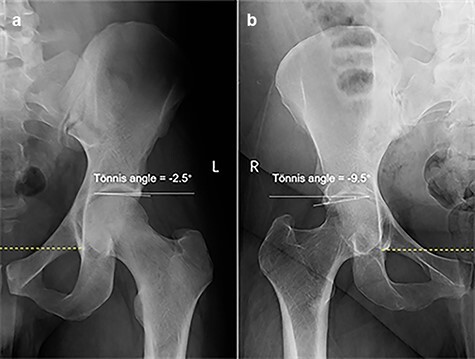
Tönnis angle << 0° in male (a) and female (b).

A thorough chart and radiographic review was performed on all patients. All radiographs were reviewed by attending- or senior resident–level orthopedic surgeons (H.P.M. and J.Z.). The first 100 radiographic reviews were evaluated by the two authors (H.P.M. and J.Z.) to ensure agreement. All patients underwent anteroposterior (AP) pelvic view (Fig. 1a and b) and lateral view (cross-table, frog-leg or 45° Dunn) radiographs upon initial assessment by a physician for hip pain. We evaluated for a pistol grip deformity on a standard AP pelvic view (Fig. 2a and b) and measured the alpha angle manually on a lateral radiographic view (cross-table, frog-leg or 45° Dunn) (Fig. 2c and d). We evaluated the COS (Fig. 3a and b), PWS (Fig. 3c and d), ISS (Fig. 3e and f), coxa profunda (Fig. 3g and h) and protrusio acetabuli (Fig. 4a and b) and measured the LCEA (Fig. 5a and b) and Tönnis angle (Fig. 6a and b) on a standard AP pelvic view. All methods of evaluation and measurement were described in a previous study [35]. Clohisy *et al*. [22] defined the standardized radiographic parameters that were used in this study. Radiographs not compliant with the parameters were not included.

The radiographic findings associated with the three types of FAI were the following:

- Cam type: typical pistol grip deformity and/or alpha angle >55° [21, 23, 24, 26].
- Pincer type: COS [22–25, 27] and/or coxa profunda or protrusio acetabuli [7, 22, 25, 27] and/or LCEA ≥40° [7, 23, 25–27] and/or Tönnis angle <0° [22].
- Mixed type: both cam and pincer type features.

### Statistical analysis

Data were collected in a password-protected database. Chi-square Test, Fisher’s exact test and independent-sample T-test were performed on the quantity and percentage of all radiographic signs and parameters between male and female. Statistical significance was considered at the 0.05 level of probability. Analyses were performed using IBM SPSS Statistics Version 25 (IBM Corporation, Armonk, NY).

## RESULTS

A total of 374 (449 hips; mean age, 28.6 ± 8.6) male patients of 612 (695 hips) and 771 (922 hips; mean age, 29.0 ± 8.4) female patients of 1281 (1447 hips) presenting with hip pain had radiographic features consistent with FAI criteria (Table I).

**Table I.** Prevalence of radiographic FAI deformities in males and females

	*n (%)*			
*Diagnosis*	*Male*	*Female*	*χ^2^*	*P*
Femoroacetabular impingement	374/612 (61.1)	771/1281 (60.2)	0.148	0.701

The distribution of various types in these FAI patients is shown in Table II. There were 429 (94 + 335) hips with cam features (cam + mixed) and 355 (20 + 335) hips with pincer features (pincer + mixed) in males and 697 (45 + 652) hips with cam features (cam + mixed) and 877 (225 + 652) hips with pincer features (pincer + mixed) in females.

**Table II.** Distribution of the three types of femoroacetabular impingement

	*n (%)*			
*Type*	*Male*	*Female*	*χ^2^*	*P*
Cam	94/449 (20.9)	45/922 (4.9)	85.427	**0.000**
Pincer	20/449 (4.5)	225/922 (24.4)	81.876	**0.000**
Mixed	335/449 (74.6)	652/922 (70.7)	2.271	0.132

Bold signifies statistical significance.

The prevalence of specific cam-type radiographic parameters is reported in Table III.

**Table III.** Cam-type radiographic findings in male and female

	*n (%) or angle (°)*			
*Signs and parameters*	*Male*	*Female*	*χ^2^/t*	*P*
Typical pistol grip deformity	328/449 (73.1)	249/922 (27.0)	262.65	**0.000**
Alpha angle >55°	399/449 (88.9)	670/922 (72.7)	46.116	**0.000**
Mean alpha angle	66.8° ± 12.2° (*n* = 449)	59.6° ± 14.9° (*n* = 922)	8.991	**0.000**

Bold signifies statistical significance.

The prevalence of specific cam-type radiographic parameters is reported in Table IV. Findings with a prevalence of >50% are listed in order as follows: COS, PWS and ISS in males and COS, coxa profunda, ISS and PWS in females.

**Table IV.** Pincer-type radiographic findings in male and female

	*N (%)*			
*Signs and parameters*	*Male*	*Female*	*χ^2^*	*P*
Crossover sign	339/449 (75.5)	723/922 (78.4)	1.470	0.225
Posterior wall sign	281/449 (62.6)	483/922 (52.4)	12.727	**0.000**
Ischial spine sign	258/449 (57.5)	507/922 (55.0)	0.748	0.387
Coxa profunda	138/449 (30.7)	706/922 (76.6)	268.106	**0.000**
Protrusio acetabuli	1/449 (0.2)	6/922 (0.7)	–	0.437^a^
Tönnis angle <0°	81/449 (18.0)	231/922 (25.1)	8.451	**0.004**
Lateral center edge angle ≥40°	54/449 (12.0)	116/922 (12.6)	0.086	0.770

a Fisher’s exact test.

Bold signifies statistical significance.

## DISCUSSION

An alpha angle >55° was the most common cam-type finding, and COS was the most common pincer type finding in both male and female patients. Additionally, the overall prevalence of radiographic findings consistent with FAI in male and female patients was 61.1% and 60.2%, respectively.

In the present investigation, mixed type was most common, which is consistent with previous studies [4, 19, 38–40]. Cam-type FAI was more common in male patients, while pincer type was more common in female patients. These findings are supported by the current literature which suggests that structural abnormalities in male FAI patients are more commonly present on the femur, while those in female FAI patients are more commonly present on the acetabulum [19, 38, 39, 41, 42].

A pistol grip deformity is a lateral osseous bump on the femoral head–neck junction (FHNJ) [43]. An increased alpha angle represents a prominence of the anterior FHNJ, which causes impingement of the anterior/anterosuperior femoral head–neck against the acetabulum [44]. Both a pistol grip deformity and an alpha angle >55° are associated with cam-type FAI [29, 45]. In the current investigation, more than two-thirds of male cam-type FAI patients (73.1%) had typical pistol grip deformities, while less than one-third of female cam-type FAI patients (27.0%) had pistol grip deformities. More male patients had an alpha angle >55° (88.9% versus 72.7%), and the mean alpha angle (66.8° ± 12.2° versus 59.6° ± 14.9°) was larger than that of female patients. Hooper *et al*. [34]. also found that cam deformities are more common and severe in male patients. In the present study, an increased alpha angle was more common than a pistol grip deformity in males and females.

Radiographic findings of COS and ISS were the first and third most common features in both male and female pincer hips. However, PWS was the second most common in male pincer hips, while coxa profunda was the second most common finding in female pincer hips. COS and PWS were first described by Reynolds *et al.* [46] in 1999, and the ISS was first described by Kalberer *et al.* [47] in 2008. These three signs can be used to diagnose acetabular retroversion, resulting in a prominent anterolateral edge of the acetabulum and potential anterolateral over-coverage [7, 48, 49]. Accordingly, these findings support the notion that the anterolateral over-coverage caused by acetabular retroversion might be the primary factor of pincer FAI in both males and females.

The prevalence of coxa profunda in pincer FAI was the fourth highest in males and the second highest in females. Both coxa profunda and protrusio acetabuli result in increased acetabular depth, causing global acetabular over-coverage [27, 50]. The deep socket potentially limits the movement of the femoral head in all directions and leads to a more circumferential pattern of impingement [38]. The presented findings suggest that global acetabular over-coverage is less common in pincer FAI than focal acetabular over-coverage resulting from acetabular retroversion. The prevalence of coxa profunda in males was less than that in females (30.7% versus 76.6%).

Both LCEA ≥40° and Tönnis angle <0° are indicators of superolateral acetabular over-coverage [51] and can be used to evaluate pincer-type FAI. In this study, the prevalences of an LCEA ≥40° and a Tönnis angle <0° in pincer FAI were 12.0% and 18.0% in males and were 12.6% and 25.1% in females, respectively. The relatively low prevalence of LCEA ≥40° and a Tönnis angle <0° suggests that the superolateral acetabular over-coverage is a less common cause of pincer FAI in both males and females.

The current investigation is not without limitations. The retrospective nature of the study affords inherent limitations. Dunn, cross-table and frog-leg lateral views were all used to measure alpha angles, but the measurement of alpha angles using all of these views has not been validated. In addition, only radiographs were evaluated so we were not able to identify cartilage and/or labral pathology. We were not able to confirm that all patients had symptoms and physical examination findings consistent with FAI. No treatment- or patient-reported outcomes were obtained in this study so we are unable to comment on the clinical relevance of each of these radiographic parameters. Despite these limitations, this study involves a large cohort of patients presenting to their physician with hip pain, which is unique. We are unaware of a prior study that reports the difference in prevalence based on the sex of FAI radiographic abnormalities in patients presenting to their physician with hip pain. Prior studies have evaluated smaller cohorts of asymptomatic patients.

## CONCLUSION

The overall prevalence of radiographic findings consistent with FAI in male and female patients with hip pain was 61.1% and 60.2%, respectively. Among the three types of FAI, mixed type was the most prevalent. The most common radiographic finding for cam-type FAI was an alpha angle >55°, and the most common radiographic finding for pincer-type FAI was a COS. Male patients were found to have a higher prevalence of cam-type deformities, whereas female patients were found to have a higher prevalence of pincer-type deformities.

## Data Availability

The data underlying this article are available in the article and in its online supplementary material.
